# Emotional and Organizational Factors in Nurse Well‐Being: A Cross‐Sectional Study

**DOI:** 10.1155/jonm/3347543

**Published:** 2026-10-19

**Authors:** Carolyn S. Phillips, Cara C. Young, Miranda Crawford, Kimberly A. Lewis, Shelli R. Kesler

**Affiliations:** ^1^ School of Nursing The University of Texas at Austin Austin Texas USA utexas.edu; ^2^ Joe C. Wen School of Nursing University of California, Los Angeles Los Angeles California USA ucla.edu

## Abstract

**Aim:**

Assess well‐being and burnout among nurses at a large acute‐care medical center and examine emotional labor and emotional intelligence factors associated with nurse well‐being.

**Background:**

Nurse burnout threatens retention, care quality, and organizational performance globally. Although staffing and workload are established contributors, the influence of emotional labor and emotional intelligence is less understood.

**Method(s):**

A cross‐sectional survey was conducted from November 2024 to May 2025 at a 435‐bed acute‐care medical center in Central Texas, United States. Registered nurses (*n* = 103) completed validated measures of well‐being, emotional labor, and emotional intelligence. We conducted descriptive statistics, regression analyses, and qualitative content analysis of open‐ended responses.

**Results:**

Poorer well‐being was significantly associated with lower emotional intelligence, particularly self‐control, and higher emotional labor demands. The regression model was statistically significant and explained 28% of the variance in Well‐Being Index scores, where higher scores indicate poorer well‐being (adjusted *R* ^2^ = 0.277); statistically significant predictors were lower emotional control (*β* = −0.290, 95% CI [–0.49, −0.09], *p* = 0.004), lower self‐control (*β* = −0.333, 95% CI [–0.56, −0.11], *p* = 0.004), and higher emotionality (*β* = 0.250, 95% CI [0.03, 0.47], *p* = 0.025). Nurses described feeling overworked and underappreciated because of staffing shortages, inequitable compensation, and emotional demands.

**Conclusion(s):**

Nurse well‐being is influenced by both emotional processes and workplace conditions. Improvement strategies should combine organizational change with interventions that strengthen emotional regulation skills.

**Implications for Nursing Management:**

Nurse managers should implement routine well‐being assessments, safe staffing, equitable recognition, and development programs supporting nurses’ perceived capacity to regulate stress and manage emotions. This study identifies modifiable predictors of nurse well‐being, informing retention and healthier work environments.

## 1. Introduction

Burnout among nurses and healthcare staff remains a persistent and escalating challenge in hospital settings, with significant implications for workforce well‐being, patient safety, and healthcare system sustainability. Burnout is a syndrome resulting from chronic workplace stress that is inadequately managed, characterized by emotional exhaustion, cynicism or detachment from work, and reduced professional efficacy [26]. Nurses are especially vulnerable due to high job demands, emotional labor, staffing shortages, and continuous exposure to patient suffering. These challenges intensified during the COVID‐19 pandemic, when nearly half of nurses reported elevated stress and sleep disturbances, and over one‐third experienced symptoms of anxiety and depression [2].

Concerns about nurse burnout predate the pandemic. Before 2020, national and international leaders warned of an impending crisis in healthcare worker well‐being, including increasing rates of psychological distress and suicide among clinicians [8, 16, 17]. In response, the National Academy of Medicine (NAM) released the *National Plan for Health Workforce Well-Being* (2024), emphasizing systems‐level responsibility for workforce well‐being and advocating routine assessment, supportive work environments, and evidence‐based organizational strategies (NAM, 2024). Despite these recommendations, many healthcare organizations continue to employ fragmented or episodic approaches to assessing burnout and well‐being, limiting their capacity to implement targeted, sustainable solutions.

Extensive evidence demonstrates that work environment characteristics strongly correlate with nurse burnout. Excessive workload, inadequate staffing, limited leadership support, and poor interprofessional collaboration have consistently been linked to higher burnout, dissatisfaction, and intent to leave [25]. Large observational studies of Magnet‐recognized hospitals reveal that approximately half of nurses report high burnout levels and that nurse well‐being is closely associated with patient safety culture and turnover intentions [1]. Notably, nurses consistently prioritize organizational interventions—such as adequate staffing, protected breaks, and improved team communication—as more effective for well‐being than individual‐focused coping strategies alone.

Systematic reviews indicate that both organizational‐ and individual‐level interventions can positively affect clinician well‐being by reducing burnout, stress, anxiety, and depressive symptoms while enhancing engagement and quality of life [7]. However, many intervention studies are limited by inconsistent use of validated measures, absence of longitudinal follow‐up, and insufficient focus on emotional labor and emotion regulation processes central to nursing practice. Emerging evidence suggests that routine unit‐level assessment of well‐being and emotional demands may be a critical initial step in identifying actionable targets for organizational change.

Evidence from demanding clinical care environments further demonstrates that nurses’ professional quality of life is shaped not only by individual coping resources but also by the broader conditions in which care is delivered. For example, nurses working in neonatal and intensive care units found that more favorable practice environments were associated with greater compassion satisfaction and lower burnout and secondary traumatic stress, while staffing and resource adequacy remained persistent areas of concern [3, 5]. These findings reinforce the importance of staffing adequacy, leadership responsiveness, interprofessional collaboration, and emotional support in settings characterized by sustained clinical intensity.

Conceptually, emotional labor and emotional intelligence, while related, reflect different aspects of nurses’ emotional work. Emotional labor refers to the occupational demand of managing felt or displayed emotions to meet professional and organizational expectations during patient care, particularly in emotionally charged interactions with patients, families, and colleagues (Hochschild, 1979). Emotional intelligence, in contrast, reflects a person’s perceived capacity to understand, use, and regulate emotion [13]. Thus, emotional labor captures what the work requires of nurses, whereas emotional intelligence reflects regulatory resources nurses may draw upon when responding to those demands. This distinction guided the present study by positioning emotional labor as a work‐related emotional demand and emotional intelligence as a potential protective or vulnerability‐related resource shaping nurse well‐being.

Accordingly, this quality improvement initiative used a prospective, cross‐sectional survey design to assess current levels of well‐being and burnout among nurses at a large acute‐care medical center in Central Texas using validated measures of well‐being, emotional labor, and emotional intelligence. The study was guided by an emotion regulation framework, which suggests that occupational emotional demands and individual regulatory capacities jointly influence psychological well‐being, while recognizing that these processes occur within broader organizational conditions such as staffing, leadership support, and resource adequacy. The specific objectives were to (1) characterize nurse well‐being levels across units; (2) examine emotional labor and emotional intelligence factors associated with well‐being; and (3) provide a replicable assessment model to inform organizational strategies supporting nurse resilience, job satisfaction, and workforce sustainability.

## 2. Methods

### 2.1. Design and Setting

This quality improvement initiative utilized a prospective, cross‐sectional survey design to assess nurse well‐being and burnout at a 435‐bed acute‐care medical center in Central Texas. The organization employs approximately 850 registered nurses across inpatient and outpatient units. The 103 completed surveys represented an approximate response proportion of 12.1% of eligible registered nurses. The project aimed to provide organizational leadership with actionable data to guide workforce well‐being initiatives and future quality improvement efforts. Although conducted for quality improvement purposes, procedures were aligned with observational research reporting standards, including anonymous survey administration, use of validated instruments, and planned quantitative and qualitative analyses. This study adheres to the Strengthening the Reporting of Observational Studies in Epidemiology (STROBE) guidelines [27]. This study was approved by the University of Texas at Austin Institutional Review Board (STUDY00006478). The study was conducted from November 2024 through May 2025.

### 2.2. Participants and Recruitment

All registered nurses employed at the medical center during the data collection period were eligible to participate. Participation was voluntary. Nurses received invitations to complete the survey via organization‐wide emails, posted flyers in clinical areas, and word of mouth. No incentives were offered. Survey responses were collected anonymously, with no identifying information obtained. Because responses were anonymous and voluntary, individual nonrespondents could not be characterized or compared with respondents.

### 2.3. Instruments

Well‐being, emotional labor, and emotional intelligence were assessed using validated self‐report instruments commonly employed in nursing and healthcare populations. Well‐being was measured using the Well‐Being Index (WBI), a brief, validated screening tool designed to assess distress and well‐being among healthcare professionals. The WBI captures multiple dimensions of occupational distress, including burnout, fatigue, quality of life, and work–life integration, with higher scores indicating poorer well‐being. The WBI has demonstrated strong psychometric properties across diverse clinician populations, with internal consistency reliability estimates ranging from Cronbach’s *α* = 0.82 to 0.89 [9, 10]. In this study, the WBI score was calculated as the sum of well‐being subscale items.

Emotional labor was assessed using the Emotional Labor Scale for Nurses, which evaluates how nurses regulate and manage emotional expression in professional interactions. The scale comprises three subdimensions: Emotional Control (efforts to manage internal emotional responses), Emotional Suppression (inhibition of genuine emotional expression), and Emotional Pretense (displaying emotions not genuinely felt). Prior studies have reported acceptable to strong internal consistency for the overall scale and subscales, with Cronbach’s *α* values ranging from 0.70 to 0.88 across nursing samples [6, 14]. In this study, Emotional Control was operationalized as the mean of items 1–7, Emotional Suppression as the mean of items 8–12, and Emotional Pretense as the mean of items 13–16.

Emotional intelligence was measured using the Trait Emotional Intelligence Questionnaire–Short Form (TEIQue–SF), which assesses stable emotional self‐perceptions across four domains: well‐being, self‐control, emotionality, and sociability. The TEIQue–SF is widely used in healthcare and occupational research and demonstrates strong internal consistency, with Cronbach’s *α* values ranging from 0.86 to 0.90 for the global score and acceptable reliability for individual subscales [19, 20].

### 2.4. Data Collection Procedures

The survey was administered electronically using an organization‐approved survey platform. Data collection occurred over a defined survey period. Responses were stored on secure servers in accordance with institutional policies. This project was conducted as a quality improvement initiative and did not involve the collection of identifiable data.

### 2.5. Quantitative Data Analysis

Descriptive statistics summarized participant characteristics and survey responses. Multiple linear regression was conducted with the WBI as the outcome and predictors including Emotional Intelligence subscales (Well‐Being, Self‐Control, Emotionality, and Sociability) and Emotional Labor subscales (Emotional Control, Emotional Suppression, and Emotional Pretense). Variables were selected based on the conceptual distinction between emotional labor as a work‐related emotional demand and emotional intelligence as a regulatory capacity that may protect or compromise well‐being. The WBI was defined as the sum of well‐being subscale scores. Emotional Control, Emotional Suppression, and Emotional Pretense were defined as the mean scores of Emotional Labor items 1–7, 8–12, and 13–16, respectively. The regression model was evaluated for normality, homoscedasticity, and outliers using standard diagnostic plots. Multicollinearity was assessed via visualizations and the variance inflation factor (VIF < 5). The alpha level was set at *p* < 0.05. All analyses and visualizations were performed using R Statistical Package v4.5.1 [21].

### 2.6. Qualitative Analysis

The survey included an open‐ended question inviting participants to provide additional comments on their experiences of well‐being and burnout. Responses were analyzed using qualitative descriptive content analysis to identify recurring patterns related to workload, appreciation, compensation, and emotional demands of nursing work [23]. Although the qualitative sample was limited to respondents who chose to provide free‐text comments, these data were used to contextualize survey findings rather than to generate exhaustive qualitative theory. Coding rigor was supported through iterative review of responses, development of preliminary categories, discussion of discrepant interpretations among the author team, and refinement of themes until consensus was reached. These findings contextualized and complemented the quantitative results.

## 3. Results

### 3.1. Participant Characteristics

A total of 103 nurses participated in the survey. Participants spanned a broad age range, with the largest proportion aged 40–49 years, followed by those aged 25–39 years. The sample was predominantly female and White, with a minority identifying as Hispanic. Detailed participant background characteristics are presented in Table 1.

**TABLE 1:** Background characteristics.

	Overall (*N* = 103)
Age (years)	
18–24	4 (3.9%)
25–29	19 (18.4%)
30–39	19 (18.4%)
40–49	34
50–59	16
60–69	10 (9.7%)
Prefer not to answer	1
Gender	
Male	10 (9.7%)
Female	91 (88.3%)
Prefer not to answer	2 (1.9%)
Race	
Asian	5 4.9%)
Black/AA	2 (1.9%)
White	90 (87.4%)
Prefer not to answer	6 (5.8%)
Ethnicity	
Hispanic	15 (14.6%)
Not Hispanic	82 (79.6%)
Prefer not to answer	6 (5.8%)

### 3.2. Occupational Characteristics

Participants exhibited diverse educational backgrounds and professional experience levels. Most held a bachelor’s degree, with over one‐fifth reporting graduate‐level education. Years of nursing experience varied, with the largest proportion reporting 10–20 years, and nearly half having at least 10 years in the profession. The majority were employed full‐time and worked day shifts, with night and weekend shifts also represented. Nurses came from various clinical departments, including labor and delivery/newborn care, operating rooms, critical care, and medical–surgical units. Additional occupational characteristics are summarized in Table 2.

**TABLE 2:** Occupational characteristics.

	Overall (*N* = 103)
RN education	
Associate	18 (17.5%)
Bachelor	63 (61.2%)
Graduate	22 (21.4%)
RN experience (years)	
1 to less than 2 years	8 (7.8%)
2–5	21 (20.4%)
6–10	15 (14.6%)
10–20	28 (27.2%)
20–30	20 (19.4%)
30+	11 (10.7%)
Tenure at organization (years)	
0–1	7 (6.8%)
1 to less than 2 years	14 (13.6%)
2–5 years	27 (26.2%)
6–10	14 (13.6%)
10–20	25 (24.3%)
20–30	11 (10.7%)
30+	5 (5.9%)
Employment status	
Full‐time	87 (84.5%)
Part‐time	13 (12.6%)
Per diem	3 (2.9%)
Shift (select all that apply)	
Day	79 (76.7%)
Night	24 (23.3%)
Weekend	8 (7.8%)
Department (select all that apply)	
Critical care	25
Medical surgical	14
Labor, delivery, and newborn	37
Rehab (inpatient and outpatient)	9
Emergency room	5
Operating room	30
Administrative/supervisory	2
Other	10

### 3.3. Multiple Linear Regression

Multiple linear regression examined emotional labor and emotional intelligence as predictors of well‐being. The model was statistically significant, explaining approximately 28% of the variance in WBI scores (adjusted *R* ^2^ = 0.277, *F* (7, 95) = 6.58, *p* < 0.001). As shown in Table 3, lower emotional control on the emotional labor scale and lower self‐control on the emotional intelligence scale were significantly associated with poorer well‐being, while higher emotionality was also significantly associated with higher WBI scores (*p* < 0.025).

**TABLE 3:** Coefficients for multiple linear regression predicting Well‐Being Index (WBI) (*N* = 103).

Predictor	Unstandardized B	Standardized *β*	95% CI	t‐statistic	*p* value	VIF
EL: Emotional Control	−1.70	−0.290	−0.49, −0.09	−2.94	0.004	1.37
EL: Emotional Suppression	0.493	0.159	−0.06, 0.38	1.43	0.157	1.75
EL: Emotional Pretense	0.258	0.076	−0.12, 0.27	0.756	0.452	1.42
EI: Well‐Being	−0.632	−0.213	−0.48, 0.05	−1.60	0.113	2.49
EI: Self‐Control	−1.00	−0.333	−0.56, −0.11	−2.93	0.004	1.82
EI: Emotionality	0.822	0.250	0.03, 0.47	2.28	0.025	1.70
EI: Sociability	−0.025	−0.009	−0.23, 0.21	−0.076	0.940	1.79

Abbreviations: EI, Emotional Intelligence; EL, Emotional Labor.

### 3.4. Qualitative Findings

A subset of participants (*n* = 36) provided qualitative responses in the optional free‐text field regarding their well‐being and burnout. Two primary themes emerged: (1) feeling *underappreciated* and (2) feeling overworked, with the combined effects contributing to substantial distress. Underappreciation manifested in various ways, notably the lack of equitable pay. One nurse stated, *“…I know—we all know-for a fact that our pay is not equitable or even remotely up to the standard for intensive care nurses.”* Another commented, *“Feel underappreciated and undercompensated after being a loyal employee with great reviews for almost 40 years” These* responses reflected perceptions of being exploited by a hospital system prioritizing finances over staff health and well‐being.

Feeling *overworked resulted from chronic understaffing, leading* to high patient ratios, elevated patient acuity, challenges inherent to 12 h shifts, and managing difficult patient behaviors. Several quotes illustrate this theme. One participant stated, “*The ratio of patients at day/night and no charge nurse to help is a big factor to burnout.”* Another nurse noted, *“If I wasn’t being pushed past my limits most days at work, my job would probably be more manageable.”* A nurse manager remarked, *“It’s very emotionally laborious to monitor the work of nurses who are heavily understaffed and stretched emotionally, mentally, and physically. I feel like a rope in a game of tug-of-war between patient safety and nurse capacity.”*

These themes were often intertwined, as summarized by one participant: *“Providing competitive pay = more retention = more staff = less burnout.” Another stated*, *“Decreasing the patient-to-staff ratio, and paying your RNs a living wage…the pay is horrible. Added work keeps getting added to the staff without compensation.”* Ultimately, participants recognized the “brokenness” of the healthcare system and the need for systems‐level change. One nurse remarked, *“Burnout is a real thing. The only way to solve this problem is to fix the broken healthcare system regarding patient care, rather than merely administering medications or band-aids; we should address the root problem so staff can stop carrying the burden.”*

## 4. Discussion

This quality improvement project provides a comprehensive assessment of nurse well‐being in an acute‐care hospital, revealing concerning levels of distress consistent with national and international trends. Prior research indicates that a substantial proportion of nurses experience burnout and psychological distress, with implications for patient safety, turnover, and workforce sustainability [1, 2, 25]. The current findings extend this evidence by identifying emotional labor and emotional intelligence as significant predictors of well‐being. Multivariable analyses showed that lower emotional control and lower emotional intelligence self‐control were associated with poorer well‐being, while higher emotionality correlated with increased distress. These results align with emerging evidence that emotion regulation capacity is a critical determinant of clinician well‐being and may represent an important, yet under‐addressed, mechanism underlying burnout [15, 20]. However, these regression findings should be interpreted in light of the model’s modest explanatory power. With the model explaining 28% of the variance, it is important to consider that other predictors of nurse well‐being such as staffing levels, leadership support, compensation, psychological safety, workload, and unit culture likely account for additional variance and may interact with emotional regulation processes.

Regardless, this study provides important insights into how emotional labor and emotional intelligence influence nurses’ well‐being. Lower emotional control and lower emotional intelligence self‐control were linked to poorer well‐being, suggesting that difficulties managing internal emotional responses and regulating stress increase vulnerability to burnout. These findings align with emotion regulation theory, which posits that ineffective regulation strategies—particularly suppression—are associated with increased psychological distress and emotional exhaustion [13]. Conversely, higher emotionality was associated with greater distress, potentially reflecting increased emotional sensitivity or empathic engagement without adequate regulatory resources. The direction of this association may indicate that nurses who are more attuned to emotional cues experience greater exposure to patient suffering, family distress, and moral tension, particularly when organizational supports are insufficient to help them process these demands. These findings raise the possibility that organizational conditions may moderate the relationship between emotional regulation and nurse well‐being. This distinction between regulatory capacity and occupational exposure may help explain why emotional characteristics operate differently across work environments. Prior research similarly indicates that heightened emotional demands in caregiving professions contribute to compassion fatigue and burnout when unsupported by adaptive regulation strategies [18].

The present findings also support consideration of emotional intelligence development as one component of nurse well‐being initiatives. Evidence from high‐acuity care settings indicates that emotional intelligence is positively associated with adaptive coping behaviors among neonatal intensive care nurses and with work performance among intensive care nurses [4, 12]. Related evidence among midwives in obstetrics and gynecology departments similarly links emotional intelligence with job performance in emotionally intense clinical environments [11]. Collectively, these findings suggest that emotional intelligence training may help strengthen coping, communication, stress management, and emotion regulation capacities. However, the current evidence base is largely cross‐sectional; therefore, emotional intelligence training should be framed as a promising professional development strategy rather than a stand‐alone solution for burnout. Such programs should complement—not replace—organizational interventions targeting staffing adequacy, leadership responsiveness, psychological safety, and supportive work environments.

Emotional labor should not be viewed solely as an individual burden but as a process influenced by organizational context. Qualitative findings emphasize how systemic factors—such as understaffing and lack of support—intensify emotional demands, limiting nurses’ capacity for effective regulation. The qualitative findings further suggest that strain may reflect not only work demands but also an imbalance between those demands and the resources, recognition, and support nurses receive. Organizational commitment to employee well‐being is crucial; however, most research on employee wellness focuses on individual‐level interventions. Although individual interventions such as mindfulness and cognitive behavioral therapy have effectively reduced stress, anxiety, and depression among healthcare workers, sustainable improvements in nurse well‐being require systems‐level change [16].

The NAM’s National Plan for Health Workforce Well‐Being offers a roadmap for diverse stakeholders to implement policy‐ and systems‐level changes to reverse clinician burnout trends (NAM, 2024). The plan outlines seven priority areas, including individual‐level solutions (e.g., fostering professional well‐being) and emphasizes systems‐level solutions such as creating and sustaining positive work and learning environments, investing in measurement, assessment, strategies, and research, and addressing compliance, regulatory, and policy barriers to daily work. NAM stresses that collective action is essential to reform a healthcare system where workers have operated in survival mode for too long (NAM, 2024).

Qualitative data indicated that emotional strain was unevenly distributed among nurses, concentrated in units where nurses reported being overextended and undervalued amid staffing instability, limited access to relief roles, and low perceived leadership responsiveness. In these settings, emotional regulation demands appeared continuous rather than episodic, suggesting that emotional labor intensifies when units operate at full capacity without flexibility or relief support. Prior research demonstrates that staffing adequacy and practice environment quality are strongly associated with nurse outcomes, including burnout and intent to leave, as well as patient outcomes such as mortality and adverse events [24]. These findings raise the possibility that cumulative emotional load may represent one pathway linking structural work conditions with nurse well‐being.

Magnet‐recognized hospitals have consistently demonstrated more favorable nurse work environments and are associated with lower burnout and improved patient outcomes. [22]. However, recent evidence suggests these relationships vary across clinical contexts and may differ substantially at the unit level [28]. Notably, misalignment between nurse leaders’ and frontline nurses’ perceptions of the work environment and patient safety culture correlates with higher burnout, even within high‐performing organizations [28]. The present findings contribute to this literature by showing that variation in emotional strain may persist within otherwise favorable environments, indicating that unit‐level conditions, rather than organizational designation alone, influence the daily sustainability of clinical work. Organization‐level workforce indicators may therefore obscure meaningful within‐organization variation in nurse well‐being and emotional strain.

These data suggest that Magnet designation criteria could evolve to better capture unit‐level conditions. Future standards may benefit from requiring stratification of workforce and work environment indicators at the unit level, as aggregated organizational data may obscure meaningful variation. Incorporating measures of within‐organization variability and emphasizing demonstrated responsiveness to identified areas of strain may more accurately reflect the consistency and sustainability of the clinical work environment. This approach shifts focus from overall performance to the absence of units with persistently unsustainable conditions. Future research should examine whether unit‐level distributions or trajectories of workforce strain provide greater actionable value than organization‐wide averages alone.

### 4.1. Leadership and Policy Implications

These findings have direct implications for leadership and policy. Unit‐level data on experienced strain and emotional workload can serve as a leadership tool by capturing the accumulation of demands that nurses report as exceeding their capacity to maintain safe, manageable practice. Such data should be integrated into routine operational decision‐making, enabling staffing and support adjustments in response to observed strain rather than relying solely on fixed staffing models or volume‐based assumptions. Practically, this involves treating break coverage, staffing adjustments, and brief opportunities for team‐based decompression as routine operational priorities rather than discretionary responses to visible distress. These findings suggest that workforce well‐being should be considered an organizational performance domain, with accountability embedded in leadership structures so that decisions regarding staffing, workflow, and resource allocation are evaluated not only for efficiency but also for their impact on clinical work sustainability. At the policy level, this approach requires incorporating unit‐level strain indicators into routine organizational reporting and governance structures alongside traditional quality and safety metrics. Establishing predefined thresholds for unit‐level strain may further facilitate timely intervention, ensuring that sustained strain patterns prompt operational review rather than informal escalation.

### 4.2. Study Limitations

Several limitations should be considered when interpreting these findings. First, the cross‐sectional design precludes causal inferences. Second, the study was conducted at a single acute‐care medical center, potentially limiting external validity and generalizability to other healthcare settings or regions. Third, the response proportion was low relative to the eligible nursing workforce, raising the possibility of nonresponse bias and limiting conclusions about nurses who did not participate. Fourth, all measures were self‐reported, introducing potential response bias, including social desirability, recall bias, and common‐method bias, which may have inflated observed associations among variables measured in the same survey. Additionally, voluntary participation may have resulted in selection bias, as nurses experiencing either high or low distress levels might have been more likely to respond. Finally, although the model explained a meaningful proportion of variance in well‐being, other unmeasured organizational and individual factors likely contribute to nurse burnout and warrant consideration in future research.

### 4.3. Future Research

Future research should build on these findings by employing longitudinal and multisite designs to elucidate temporal relationships among emotional labor, emotional intelligence, and nurse well‐being across diverse healthcare environments. Longitudinal studies should also examine whether organizational conditions moderate relationships among emotional demands, regulatory capacities, and nurse well‐being over time, including whether sustained exposure to high‐demand, low‐resource environments attenuates the protective effects of individual emotional‐regulation resources. Intervention studies are particularly needed to evaluate strategies targeting both emotion regulation capacities and organizational conditions, including staffing models, leadership practices, and team‐based supports. Incorporating routine unit‐level assessments of emotional labor and well‐being into quality improvement frameworks may assist organizations in identifying high‐risk areas and tailoring interventions accordingly.

## 5. Conclusion

This study highlights the critical role of emotional labor and emotional intelligence in shaping nurses’ well‐being within acute‐care settings. Findings demonstrate that emotional demands and regulatory capacities are key contributors to burnout, while qualitative data reveal the amplifying effects of organizational factors such as understaffing and perceived lack of support. Collectively, these results underscore that improving nurse well‐being requires integrated, multilevel approaches addressing both individual and system‐level factors. Embedding routine assessment of well‐being and emotional demands into organizational practice provides a practical and scalable pathway to advance healthy work environments and sustain the nursing workforce.

## Funding

This research received no specific grant from any funding agency in the public, commercial, or not‐for‐profit sectors.

## Disclosure

The author team includes a statistician (Dr. Shelli Kesler).

## Ethics Statement

This study was approved by the University of Texas at Austin Institutional Review Board.

## Consent

This study was reviewed and determined to be exempt by the institutional review board, and a waiver of informed consent was granted.

## Conflicts of Interest

The authors declare no conflicts of interest.

## Data Availability

Data supporting the findings of this study are available from the corresponding author upon reasonable request. The data are not publicly available due to institutional review board restrictions and the potential for identification of participants.

## References

[1] Aiken L. H. , Lasater K. B. , Sloane D. M. et al., Physician and Nurse Well-Being and Preferred Interventions to Address Burnout in Hospital Practice: Factors Associated With Turnover, Outcomes, and Patient Safety, JAMA Health Forum. (2023) 4, no. 7, 10.1001/jamahealthforum.2023.1809. PMC10329209 37418269

[2] Al Maqbali M. , Al Sinani M. , and Al-Lenjawi B. , Prevalence of Stress, Depression, Anxiety and Sleep Disturbance Among Nurses During the COVID-19 Pandemic: A Systematic Review and meta-analysis, Journal of Psychosomatic Research. (2021) 141, 10.1016/j.jpsychores.2020.110343. PMC7831768 33360329

[3] Ayed A. , Abu Ejheisheh M. , Aqtam I. , Batran A. , and Farajallah M. , The Relationship Between Professional Quality of Life and Work Environment Among Nurses in Intensive Care Units, Inquiry: A Journal of Medical Care Organization, Provision and Financing. (2024) 61, 10.1177/00469580241297974. PMC11550503 39520216

[4] Ayed A. , The Relationship Between Emotional Intelligence and Coping Behaviors Among Nurses in the Neonatal Intensive Care Unit, SAGE Open Nursing. (2025) 11, 10.1177/23779608251330097. PMC11920997 40109576

[5] Batran A. , Aqtam I. , Ayed A. , and Ejheisheh M. A. , The Relationship Between Professional Quality of Life and Work Environment Among Nurses in Neonate Care Units, PLoS One. (2025) 20, no. 4, 10.1371/journal.pone.0322023. PMC12027106 40279366

[6] Brotheridge C. M. and Lee R. T. , Development and Validation of the Emotional Labour Scale, Journal of Occupational and Organizational Psychology. (2003) 76, no. 3, 365–379, 10.1348/096317903769647229.

[7] Cohen C. , Pignata S. , Bezak E. , Tie M. , and Childs J. , Workplace Interventions to Improve Well-Being and Reduce Burnout for Nurses, Physicians and Allied Healthcare Professionals: A Systematic Review, BMJ Open. (2023) 13, no. 6, 10.1136/bmjopen-2022-071203. PMC10314589 37385740

[8] Davidson J. E. , Proudfoot J. , Lee K. , Terterian G. , and Zisook S. , A Longitudinal Analysis of Nurse Suicide in the United States (2005–2016) With Recommendations for Action, Worldviews on Evidence-Based Nursing. (2020) 17, no. 1, 6–15, 10.1111/wvn.12419. 32017434

[9] Dyrbye L. N. , Johnson P. O. , Johnson L. M. et al., Efficacy of the Well-Being Index to Identify Distress and Stratify Well-Being in Nurse Practitioners and Physician Assistants, Journal of the American Association of Nurse Practitioners. (2019) 31, no. 7, 403–412, 10.1097/JXX.0000000000000179. 30829967

[10] Dyrbye L. N. , Massie F. S. , Eacker A. et al., Relationship Between Burnout and Professional Conduct and Attitudes Among US Medical Students, The Journal of the American Medical Association. (2010) 304, no. 11, 1173–1180, 10.1001/jama.2010.1318. 20841530

[11] Eqtait F. A. , Ayed A. J. , Anabtawi R. et al., The Relationship Between Emotional Intelligence and Work Performance Among Palestinians’ Midwives in Obstetrics and Gynecology Departments, Scientific Reports. (2026) 16, no. 1, 10.1038/s41598-026-35463-z. PMC12876855 41526638

[12] Fashafsheh I. H. , Eqtait F. A. , Hammad B. M. , Ayed A. J. , and Salameh B. S. , The Impact of Emotional Intelligence on Work Performance Among ICU Nurses in Palestine: A Cross-Sectional Study, BMC Nursing. (2025) 24, no. 1, 10.1186/s12912-025-03068-y. PMC11993981 40221734

[13] Gross J. J. , Emotion Regulation: Current Status and Future Prospects, Psychological Inquiry. (2015) 26, no. 1, 1–26, 10.1080/1047840X.2014.940781.

[14] Lee R. T. and Brotheridge C. M. , Words From the Heart Speak to the Heart, Career Development International. (2011) 16, no. 4, 401–420, 10.1108/13620431111158805.

[15] Melnyk B. M. , Kelly S. A. , Stephens J. et al., Interventions to Improve Mental Health, Well-Being, Physical Health, and Lifestyle Behaviors in Physicians and Nurses: A Systematic Review, American Journal of Health Promotion. (2020) 34, no. 8, 929–941, 10.1177/0890117120920451. PMC8982669 32338522

[16] Melnyk B. M. , Burnout, Depression and Suicide in Nurses/Clinicians and Learners: An Urgent Call for Action to Enhance Professional Well-being and Healthcare Safety, Worldviews on Evidence-Based Nursing. (2020) 17, no. 1, 2–5, 10.1111/wvn.12416. 32017437

[17] Taking Action Against Clinician Burnout: A Systems Approach to Professional Well-Being, 2019, The National Academies Press, 10.17226/25521. 31940160

[18] Partlak Günüşen N. , Şengün İnan F. , Üstün B. , Serttaş M. , Sayin S. , and Yaşaroğlu Toksoy S. , The Effect of a Nurse-Led Intervention Program on Compassion Fatigue, Burnout, Compassion Satisfaction, and Psychological Distress in Nurses: A Randomized Controlled Trial, Perspectives in Psychiatric Care. (2022) 58, no. 4, 1576–1586, 10.1111/ppc.12965. 34706071

[19] Petrides K. V. and Furnham A. , Trait Emotional Intelligence: Psychometric Investigation With Reference to Established Trait Taxonomies, European Journal of Personality. (2001) 15, no. 6, 425–448, 10.1002/per.416.

[20] Petrides K. V. , Parker J. D. A. , Saklofske D. H. , and Stough C. , Psychometric Properties of the Trait Emotional Intelligence Questionnaire (Teique), Assessing Emotional Intelligence, 2009, Springer, 85–101, 10.1007/978-0-387-88370-0_5.

[21] Core Team R. , R: A Language and Environment for Statistical Computing, 2025, R Foundation for Statistical Computing.

[22] Rodríguez-García M. C. , Márquez-Hernández V. V. , Belmonte-García T. , Gutiérrez-Puertas L. , and Granados-Gámez G. , Original Research: How Magnet Hospital Status Affects Nurses, Patients, and Organizations: A Systematic Review, American Journal of Nursing. (2020) 120, no. 7, 28–38, 10.1097/01.NAJ.0000681648.48249.16. 32541337

[23] Sandelowski M. , Whatever Happened to Qualitative Description?, Research in Nursing & Health. (2000) 23, no. 4, 334–340, 10.1002/1098-240X(200008)23:4<334::AID-NUR9>3.0.CO;2-G. 10940958

[24] Schlak A. E. , Aiken L. H. , Chittams J. , Poghosyan L. , and McHugh M. , Leveraging the Work Environment to Minimize the Negative Impact of Nurse Burnout on Patient Outcomes, International Journal of Environmental Research and Public Health. (2021) 18, no. 2, 10.3390/ijerph18020610. PMC7828279 33445764

[25] Shah M. K. , Gandrakota N. , Cimiotti J. P. , Ghose N. , Moore M. , and Ali M. K. , Prevalence of and Factors Associated With Nurse Burnout in the US, JAMA Network Open. (2021) 4, no. 2, 10.1001/jamanetworkopen.2020.36469. PMC7862989 33538823

[26] Sullivan D. , White K. M. , and Frazer C. , Factors Associated With Burnout in the United States Versus International Nurses, Nursing Clinics of North America. (2022) 57, no. 1, 29–51, 10.1016/j.cnur.2021.11.003. 35236607

[27] von Elm E. , Altman D. G. , Egger M. et al., The Strengthening the Reporting of Observational Studies in Epidemiology (STROBE) Statement: Guidelines for Reporting Observational Studies, Annals of Internal Medicine. (2007) 147, no. 8, 573–577, 10.7326/0003-4819-147-8-200710160-00010. 17938396

[28] Yu H. , Aiken L. H. , Grischott L. J. et al., Differences in Hospital Staff Nurses’ and Nurse Leaders’ Assessments of the Work Environment and Patient Safety are Associated With Staff Nurse Burnout, Nursing Outlook. (2025) 73, no. 6, 10.1016/j.outlook.2025.102548. PMC12699971 40946434

